# Retraction: A novel primary stability test method for artificial acetabular shells considering vertical load during level walking and shell position

**DOI:** 10.1371/journal.pone.0350164

**Published:** 2026-05-26

**Authors:** The PLOS One Editors

Following publication of [1], concerns were raised that this article contains a substantial degree of overlap with an article previously published in a Japanese-language journal [2] by the same authors, including contents of the Introduction, Materials and methods, and Discussion sections, Fig 2, Figs 6-10, and quantitative results.

On the basis of PLOS’ editorial assessment, the *PLOS One* Editors consider the extent of overlap is such that this article [1] constitutes a redundant publication. The corresponding author acknowledged that there was substantial overlap between [1] and [2] and apologized that the related work was not declared at the time of submission.

The *PLOS One* Editors retract this article [1] because, per our editorial assessment, it does not meet *PLOS One*’s second publication criterion [3].

All authors agreed with the retraction.

The retracted article [1] was removed from the *PLOS One* website at the time of retraction, due to the similarities with [2]. The article’s Copyright statement, Data Availability statement, and Peer Review History were also updated at that time, and the removed contents are no longer offered under the Creative Commons Attribution License.
